# phyloMDA: an R package for phylogeny-aware microbiome data analysis

**DOI:** 10.1186/s12859-022-04744-5

**Published:** 2022-06-06

**Authors:** Tiantian Liu, Chao Zhou, Huimin Wang, Hongyu Zhao, Tao Wang

**Affiliations:** 1 SJTU-Yale Joint Center for Biostatistics and Data Science, Shanghai Jiao Tong University, Shanghai, China; 2 Department of Biostatistics and Bioinformatics, Duke University School of Medicine, Durham, North Carolina USA; 3 Department of Biostatistics, Yale University, New Haven, Connecticut USA; 4 Department of Statistics, School of Mathematical Sciences, Shanghai Jiao Tong University, Shanghai, China; 5 MoE Key Lab of Artificial Intelligence, AI Institute, Shanghai Jiao Tong University, Shanghai, China

**Keywords:** Phylogeny-aware analysis, Relative abundances, Multivariate model

## Abstract

**Background:**

Modern sequencing technologies have generated low-cost microbiome survey datasets, across sample sites, conditions, and treatments, on an unprecedented scale and throughput. These datasets often come with a phylogenetic tree that provides a unique opportunity to examine how shared evolutionary history affects the different patterns in host-associated microbial communities.

**Results:**

In this paper, we describe an R package, phyloMDA, for phylogeny-aware microbiome data analysis. It includes the Dirichlet-tree multinomial model for multivariate abundance data, tree-guided empirical Bayes estimation of microbial compositions, and tree-based multiscale regression methods with relative abundances as predictors.

**Conclusion:**

phyloMDA is a versatile and user-friendly tool to analyze microbiome data while incorporating the phylogenetic information and addressing some of the challenges posed by the data.

**Supplementary Information:**

The online version contains supplementary material available at 10.1186/s12859-022-04744-5.

## Background

Advances in high-throughput sequencing technologies are allowing large-scale profiling of microbial communities. After quality control and data preprocessing, sequencing reads are organized into tables or matrices, in which the rows represent samples and the columns are counts of clustered sequences that represent community members (such as operational taxonomic units or amplicon sequence variants). In many microbial survey studies, there is also a phylogenetic tree that depicts the evolutionary relationships among microbes based on their genetic closeness, and a metadata matrix that contains information about the samples (such as body mass index or disease status).

Data from microbiome studies have proven useful for understanding the important role of microbes in human health and disease. However, analyzing and interpreting these data is challenging due to high dimensionality, uneven sequencing depth, data sparsity, and compositionality [1]. Apart from these challenges, there is an increasing need and unique opportunity to develop methods that efficiently leverage information on the phylogenetic relationships among taxa [2–4]. Although statistical and machine learning approaches have been developed and publicly available to address some of the challenges, they typically ignore the phylogenetic tree. Here, we propose an R package, phyloMDA, for phylogeny-aware microbiome data analysis.

## Implementation

**Fig. 1:**
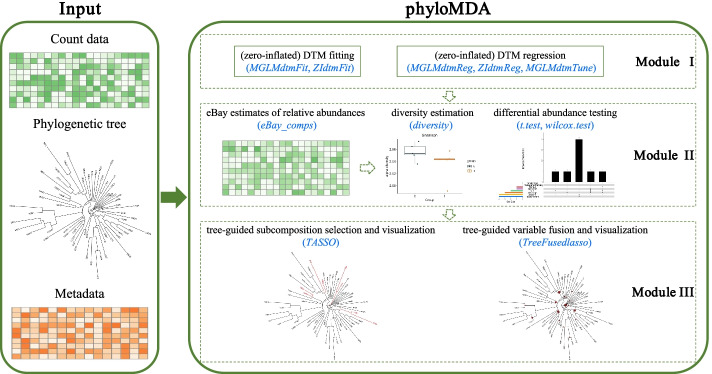
Chart illustrating the three modules of the phyloMDA package proposed here for phylogeny-aware microbiome data analysis. phyloMDA requires three input files: a count matrix, a metadata matrix, and a phylogenetic tree. Module I contains R functions for fitting (zero-inflated) Dirichlet-tree multinomial models for multivariate abundance data. Some of these functions are invoked in Module II to produce tree-guided empirical Bayes (eBay) estimates of microbial compositions. These relative abundances are then used as input into high-level analyses. In particular, Module III contains R functions for tree-based multiscale regressions with relative abundances as predictors

phyloMDA takes as input a count matrix, a metadata matrix, and a phylogenetic tree. It consists of three modules: multivariate modeling of microbial counts, extraction of relative abundances from counts, and regression with relative abundances as predictors (Fig. 1). A user manual is provided in Additional file 1.

### Multivariate modeling of microbial counts

Module I assumes that the multivariate count data are distributed according to Dirichlet-tree multinomial (DTM) [5, 6]. Loosely speaking, DTM is the product of Dirichlet multinomials that factorize over the phylogenetic tree. The log link function is used to link parameters of DTM to covariates. Parameters of the DTM model are estimated by maximum likelihood or penalized maximum likelihood. Extensions to the zero-inflated DTM model [7] are also implemented.

### Extraction of relative abundances from counts

Module II of the package applies an empirical Bayes strategy to estimate the relative abundances underlying raw count data [7, 8]. By specifying a multinomial distribution for multivariate counts and a prior for its probability vector, the posterior mean provides a natural estimate of relative abundances. The empirical Bayes procedure assumes a (zero-inflated) Dirichlet-tree prior and estimates the unknown hyper-parameters by maximizing the data evidence, which amounts to fitting a (zero-inflated) DTM model (Module I).

Extracted relative abundances, known as compositions, are used as input into downstream analyses such as diversity estimation, differential abundance testing, and compositionally aware data analysis.

### Regression with relative abundances as predictors

Linear log-contrast models are popular for regressing a univariate response on a compositional predictor [9]. [10] introduce the concept of subcomposition selection and illustrate that, under the linear log-constrast model, component selection outputs a single subcomposition composed of selected components. They also develop a multiscale subcomposition selection method, called tree-guided automatic subcomposition selection operator (TASSO), for selecting subcompositions at subtree levels.

Assuming that phylogenetically close taxa have similar associations with a host phenotype, [11] introduce the concept of variable fusion that is immune to zeros and is operationally adapted to the compositionality. They further propose tree-guided fused lasso under the standard linear model.

Module III implements these two procedures.

## Results

For illustration, we apply phyloMDA to the COMBO dataset [12] which, after preprocessing, consists of a matrix that relates abundances of 62 OTUs to 98 subjects, a phylogenetic tree that reflects the evolutionary relationship of these OTUs, and metadata that provides information about the subjects such as body mass index (BMI). It took about 10 minutes and 775 kilobytes to analyze the data on a Macbook Pro (Intel Core i5, 1.4 GHz, 8GB RAM).

### Modeling for multivariate abundance data

The (zero-inflated) DTM distribution can be used to model multivariate abundance data. The results of DTM fitting and regression can be found in Additional file 1.

### Estimation of microbial compositions

Microbiome data are often normalized prior to downstream analysis. By assuming a multinomial distribution for microbial counts and a Dirichlet-tree prior for the proportions, we transform raw counts into relative abundances by using the estimated posterior mean. The results of diversity estimation and differential abundance testing on extracted relative abundances can be found in Additional file 1.

### Tree-based regression with relative abundances as predictors

**Fig. 2:**
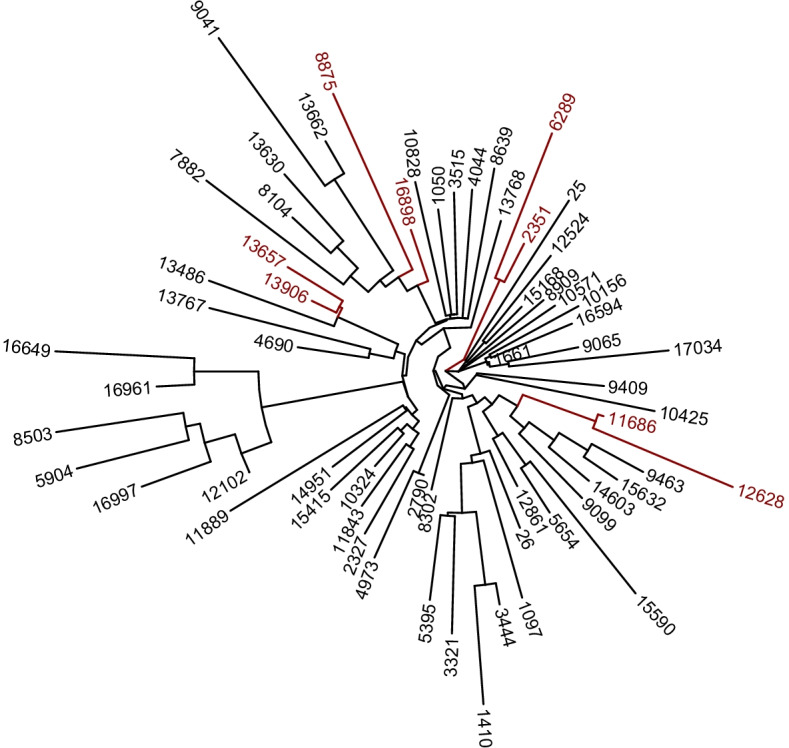
Tree visualization of the results of applying TASSO to the COMBO data, with the selected features shown in red

Since the compositions carry only relative information, subcompositions are fundamental objects of investigation in compositional data analysis. We consider the regression of BMI on the estimated composition and apply TASSO to select subcompositions at subtree levels. The results are shown in Fig. 2. We can see that TASSO detects four two-component subcompositions. We also apply tree-guided fused lasso to construct predictive models comprised of bacterial taxa at multiple taxonomic levels. The results can be found in Additional file 1.

## Conclusion

We have presented a new and simple-to-use tool, phyloMDA, that offers three modules for analyzing microbiome data while simultaneously incorporating the phylogenetic information and mitigating the challenges posed by the data, ranging from the modeling of multivariate abundance data to estimation of microbial compositions to regression of a phenotype onto relative abundances. Note that the phyloseq package has been developed for microbiome data analysis [13], and that phyloMDA takes a phyloseq object as the input file. In addition to being a tool to import, store, and graphically display phylogenetic sequencing data, phyloseq also provides convenience analysis wrappers for common analysis tasks by leveraging tools available in R for ecology and phylogenetic analysis. phyloMDA can be easily extended or integrated into pipelines for microbiome data analysis, especially in cooperating with other R packages, such as phyloseq and the compositions package [14]; the latter offers methods for compositional data analysis by providing descriptive statistics, plotting, multivariate analysis, standard transforms, and so on.

## Supplementary Information

**Additional file 1** A user manual for the phyloMDA package

## Data Availability

The package phyloMDA and example data are freely available at https://github.com/liudoubletian/phyloMDA. **Availability and requirements** Project name: phyloMDA. Project home page: https://github.com/liudoubletian/phyloMDA. Operating system(s): Platform independent. Programming language: R. Other requirements: None. License: GPL (≥2). Any restrictions to use by non-academics: No restrictions.

## Abbreviations

DTM: Dirichlet-tree multinomial
TASSO: Tree-guided automatic subcomposition selection operator
