# Supplementary material for: phyloMDA: an R package for phylogeny-aware microbiome data analysis
Source: BMC Bioinformatics. 2022 Jun 6;23:213. doi: 10.1186/s12859-022-04744-5 (PMC9169257; doi:10.1186/s12859-022-04744-5)
Supplement: Supplementary file 1 — Additional file 1 A user manual for the phyloMDA package [file 12859_2022_4744_MOESM1_ESM.html]

The supplementary for “phyloMDA : An R package for phylogeny-aware microbiome data analysis”


# The supplementary for “phyloMDA : An R package for phylogeny-aware microbiome data analysis”

#### Chao Zhou, Tiantian Liu, Huimin Wang, Hongyu Zhao, and Tao Wang

- 1. Introduction
  - 1.1 Microbiome Data
- 2. Multivariate modeling of microbial counts
  - 2.1 Dirichlet-multinomial model
  - 2.2 (Zero-inflated) Dirichlet-tree multinomial model
- 3. Extraction of relative abundances from counts
  - 3.1 Empirical Bayes normalization
  - 3.2 Phylogeny-aware normalization
  - 3.3 Statistical analysis of compositional data
  - 3.4 Diversity estimation
  - 3.5 Differential abundance analysis: An illustration
- 4. Regression with relative abundances as predictors
  - 4.1 Constrained lasso and log-ratio lasso
  - 4.2 Phylogeny-aware subcomposition selection
  - 4.4 Linear regression and variable fusion
- Reference

## 1. Introduction

This is the manual for **phyloMDA**, an R package for phylogeny-aware microbiome data analysis. phyloMDA takes as input a count matrix, a metadata matrix, and a phylogenetic tree. It consists of three modules: multivariate modeling of microbial counts, extraction of relative abundances from counts, and regression with relative abundances as predictors.


### 1.1 Microbiome Data

We illustrate statistical methods for analyzing tree structured microbiome data by applying them to the COMBO dataset from a cross-sectional study on relating dietary habits to gut microbiome [1]. The preprocessed data set consists of a matrix that relates abundances of 62 OTUs to 98 samples, a phylogenetic tree that reflects the evolutionary relationship of these OTUs, and metadata that provide information about the samples such as BMI and measurements of dietary nutrients. We will be working with the `phyloseq` object.

**Load Packages**

```
library(MGLM);
library(plyr);
library(caper);
library(genlasso);
library(magrittr);
library(foreach);
library(ape);
library(miLineage);
library(ggplot2);
library(dplyr);
library(readxl);
library(methods);
library(BiocManager);
library(phyloseq);
library(ggtree);
library(adaANCOM);
library(phyloMDA);
```

**Load Example Data**

```
(phyloseq.obj <- combo.phyloseq.obj)
```

```
## phyloseq-class experiment-level object
## otu_table()   OTU Table:         [ 62 taxa and 98 samples ]
## sample_data() Sample Data:       [ 98 samples by 231 sample variables ]
## tax_table()   Taxonomy Table:    [ 62 taxa by 9 taxonomic ranks ]
## phy_tree()    Phylogenetic Tree: [ 62 tips and 61 internal nodes ]
```

**Plot the phylogenetic tree**

```
library(ggtree); 
ggtree(phyloseq.obj@phy_tree, aes(), layout="fan", size=0.3, ladderize=F) +
  geom_tiplab(size=2, hjust=-0.05, align = F) + theme_tree()
```

**Heatmap of microbial counts**

```
plot_heatmap(phyloseq.obj, taxa.order = taxa_names(phyloseq.obj))
```

**Calculate the proportion of zeros**

```
otu_tab <- t(phyloseq.obj@otu_table@.Data)
n <- nrow(otu_tab) # sample size
K <- ncol(otu_tab) # number of taxa
zp <- sum(otu_tab==0)/n/K 
round(zp, 4) # zero proportion
```

```
## [1] 0.6141
```

**View the metadata**

```
library(DT);  # an R interface to the JavaScript library DataTables
library(magrittr);  # chaining commands %>% 
metadata <- sample_data(phyloseq.obj)
ex_nut <- metadata[, 18:20] %>% round(., 4)
cbind(metadata[, 1:6], ex_nut) %>% datatable() # here only list 3 nutrients
```

**Plot the sum of counts across samples and across taxa**

```
library(ggpubr)   # creating and customizing ggplot2- based publication ready plots
theme_sets <- theme_bw() +  theme(panel.border = element_blank(),
                                  panel.grid.major = element_blank(),
                                  panel.grid.minor = element_blank(),
                                  axis.line = element_line(colour = "black"))

pb1 <- plot_bar(phyloseq.obj) + geom_bar(stat="identity", position="stack") + 
   labs(title='Sum of counts') +  theme_sets +
  theme(axis.text.x=element_text(face="bold",size=8,angle=90))

pb2 <- plot_bar(phyloseq.obj, 'OTU',fill="phylum") +
  geom_bar(stat="identity", position="stack") + labs(title='Sum of counts')+ theme_sets +
  theme(axis.text.x=element_text(face="bold",size=8,angle=90))
ggarrange(pb1, pb2, nrow=2, ncol=1)
```

**Visualize the over-dispersion**

```
ggplot(data=NULL, aes(x=colMeans(otu_tab), y=apply(otu_tab, 2, sd))) + 
  geom_abline(slope = 1, intercept = 0) + geom_point() + 
  labs(title='Over-dispersion', x='Mean', y='SD') + theme_sets
```

## 2. Multivariate modeling of microbial counts

One distribution for describing bacterial counts is the multinomial (MN) distribution. We fit this distribution by maximum likelihood. The estimates of probabilities are sample proportions.

**View sample proportions**

```
sample_prop  <- colMeans(otu_tab/rowSums(otu_tab)) 
ggplot(data=NULL, aes(x=names(sample_prop), y=sample_prop, size=sample_prop)) +  
  theme_sets +
  theme(axis.text.x=element_text(face="bold",size=6,angle=90), legend.position  = 'none') +
  geom_vline(xintercept = names(sample_prop), linetype="dotted", color='lightgrey') +
  geom_point() +
  labs(x='OTU', y='Sample proportion')
```

In the multinomial-logit regression model, the probabilities of the MN distribution are related to a vector of predictors via the logit linear transformation [2].

**Load Packages**

```
library(MGLM) # A package for multivariate response generalized linear models
```

**Simple regression with a single predictor**

```
sodium <- metadata$sodium 
reg_mn <- MGLMreg(otu_tab ~ 1 + sodium, dist = 'MN') # MGLMfit fits various multivariate discrete distributions 
reg_mn@test # overall test
```

```
##             wald value Pr(>wald)
## (Intercept) 1322196.89         0
## sodium        22716.23         0
```

```
reg_mn@logL # fitted simple MN regression loglikelihood
```

```
## [1] -388327.3
```

We can also use `MGLM` to fit the MN distribution.

```
fit_mn <- MGLMfit(data=otu_tab, dist = 'MN')
fit_mn@logL # fitted MN loglikelihood
```

```
## [1] -401364.8
```

### 2.1 Dirichlet-multinomial model

In the presence of over-dispersion, the Dirichlet-multinomial (DM) distribution is a popular alternative to the MN distribution. It is a compound MN distribution with weights from the Dirichlet distribution. DM is MN augmented with one additional parameter (known as the over-dispersion parameter). The function **MGLMfit** can also fit this distribution.

```
fit_dm <- MGLMfit(data=otu_tab, dist='DM')
fit_dm@logL      # fitted DM loglikelihood
```

```
## [1] -13845.53
```

```
fit_dm@LRTpvalue # LRT MN vs DM
```

```
## [1] 0
```

To run a regression, the log link is used to relate the parameters to the predictors.

```
reg_dm <- MGLMreg(otu_tab~1+sodium, dist='DM')
round(reg_dm@test, 4)
```

```
##             wald value Pr(>wald)
## (Intercept) 11851.5553    0.0000
## sodium         81.4292    0.0496
```

```
reg_dm@logL
```

```
## [1] -13805.07
```

Rather than simple or multiple regression, we can also run penalized regression, which is preferred when the number of predictors (here, nutrient measurements) is comparable to or larger than the sample size. For illustration, we carry out a penaltized DM regression using the first 20 nutrients. The tuning parameters are typically chosen by an information criterion or cross-validation, here we use BIC.

```
# MGLMsparsereg fits sparse regression in multivariate generalized linear models.
# Other penalty types than “sweep” are allowed
X <-  metadata %>% as.matrix %>% .[,18:37] %>% apply(., 2, as.numeric)
spreg_dm <- MGLMtune(formula = otu_tab~1+X, dist = 'DM', penalty = 'sweep', ngridpt = 20, penidx = c(F, rep(T, 20)))
show(spreg_dm)
```

```
## Call: MGLMtune(formula = otu_tab ~ 1 + X, dist = "DM", penalty = "sweep", 
##     ngridpt = 20, penidx = c(F, rep(T, 20)))
## 
## Distribution: Dirichlet Multinomial
## Log-likelihood: -13820.67
## BIC: 27953.12
## AIC: 27777.34
## Degrees of freedom: 68
## Lambda: 37.85639
## Number of grid points: 20
```

**Tuning via BIC**

```
path_res <- spreg_dm@path
ggplot(data=path_res, aes(x=Lambda, y=BIC)) + 
  geom_point() + geom_line() + theme_sets + labs(x='lambda') + 
  geom_vline(xintercept = spreg_dm@select@lambda, color='red', linetype="dotted")
```

**View estimated coefficients**

```
spreg_coef <- coef(spreg_dm) %>% t
colnames(spreg_coef) <- c('Intercept', colnames(X))
datatable(round(spreg_coef[, 1:11], 4)) # show the first 10 components
```

### 2.2 (Zero-inflated) Dirichlet-tree multinomial model

To take into account the tree information, Wang, T. and Zhao, H. proposed Dirichlet-tree multinomial (DTM) distribution, defined to be the product of DM distributions that factorize over the phylogenetic tree [3]. Zhou, C, Zhao, H., and Wang T. extended DTM to Zero-inflated DTM (ZIDTM) [4] by the same way. Since the distributions placed on different internal nodes are independent, estimation of the parameters of the DTM model can be carried out separately.

```
tree <- phy_tree(phyloseq.obj)
fit_dtm <- MGLMdtmFit(otu_tab, tree)
(logL_dtm <- Extract_logL(fit_dtm)) # DTM loglikelihood
```

```
## [1] -13160.69
```

```
pchisq(2*(logL_dtm - fit_dm@logL), df=61*2-62, lower.tail = F)
```

```
## [1] 7.642585e-247
```

```
fit_zidtm <- ZIdtmFit(otu_tab, tree)
(logL_zidtm <- Extract_logL(fit_zidtm)) # ZIDTM loglikelihood
```

```
## [1] -13178.28
```

```
pchisq(2*(logL_zidtm - logL_dtm), df=61*2-62, lower.tail = T)
```

```
## [1] 0
```

```
reg_dtm <- MGLMdtmReg(otu_tab, sodium, tree)
Extract_logL(reg_dtm) # DTM regression loglikelihood
```

```
## [1] -13132.97
```

```
reg_zidtm <- ZIdtmReg(otu_tab, X.mean=sodium, tree=tree) # test results for ZIDTM
```

We can also perform penalized DTM regression by running penalized DM in a node-wise manner.

```
sreg_dtm <- MGLMdtmSparseReg(otu_tab,X, tree, lambda=Inf, penalty="sweep")
Extract_logL(sreg_dtm)
```

```
## [1] -13224.1
```

**Tuning via BIC one each node**

```
Extract_BIC(sreg_dtm)
```

```
## [1] 26956.14
```

**View estimated coefficients**

```
spreg_dtmcoef <- lapply(sreg_dtm, function(x) coef(x)) %>% do.call(cbind, .) %>% t
colnames(spreg_dtmcoef) <- c('Intercept', colnames(X))
datatable(round(spreg_dtmcoef[,1:11], 4)) # show the first 10 components
```

We also implement tuning version for lambda.

```
sreg_dtm_tune <- MGLMdtmTune(otu_tab, X, tree, penalty="sweep")
Extract_logL(sreg_dtm_tune) # not run
```

## 3. Extraction of relative abundances from counts

Microbiome data are often normalized prior to downstream analysis. Two widely-used normalization approaches are rarefying and total sum scaling [5]. Liu, T. et al. proposed an empirical Bayes approach for transforming raw counts into relative abundances [6].

### 3.1 Empirical Bayes normalization

Assuming a multinomial distribution for microbial counts and a Dirichlet prior for the proportions, the marginal distribution of each count vector is DM, with the same set of parameters as the prior. We estimate these parameters from OTU counts across samples by maximum likelihood. The empirical Bayes method normalizes the data using the estimated posterior mean.

```
eBay.comps <- eBay_comps(otu_tab, prior="Dirichlet")
datatable(round(eBay.comps[,1:10],4))
```

### 3.2 Phylogeny-aware normalization

We can further incorporate phylogeny into the normalization process by assuming a Dirichlet-tree prior [6]. The resulting marginal distribution of each count vector is then DTM.

```
eBay.tree.comps <- eBay_comps(otu_tab, prior="Dirichlet-tree", tree=tree)
datatable(round(eBay.tree.comps[, 1:10], 5))
```

### 3.3 Statistical analysis of compositional data

Microbiome data are compositional and should be treated as compositions. However, since compositions are constrained by the simplex, the analysis of compositional data using traditional tools can be misleading [7]. One way to address this issue is to use ratio transformations and then take the logarithm of these ratios, known as log-ratios. Often, the additive log-ratio and centered log-ratio transformations are used.

```
comps_alr <- alr_trans(eBay.comps)
comps_clr <- clr_trans(eBay.comps)
```

### 3.4 Diversity estimation

When the compositions are extracted from microbial count data, downstream analyses such as diversity estimation, and compositionally aware data analysis are easily achievable. Here, we present two types of diversity estimation: alpha diversity and beta diversity.

- alpha diversity

```
library(vegan)
meta_sam <- metadata %>% .[.$bmicat1norm2ow3ob!=2, ]
eBay.comps_sub <- eBay.comps[as.character(meta_sam$pid),]
Shannon.Wiener <- diversity(eBay.comps_sub, index = "shannon")
Simpson <- diversity(eBay.comps_sub, index = "simpson") #Simpson
Inverse.Simpson <- diversity(eBay.comps_sub, index = "inv") #Inverse Simpson
group <- c()
group[which(meta_sam$bmicat1norm2ow3ob==1)] <- "normal"
group[which(meta_sam$bmicat1norm2ow3ob==3)] <- "obese"

library(ggpubr) ###
div.sha <- data.frame(shannon = Shannon.Wiener,group= group)
pic1 <- ggboxplot(div.sha,
                  x="group",
                  y="shannon",
                  color="group",
                  palette = "jama",legend = "right",
                  add = "jitter",xlab="Group",ylab="alpha diversity",title="Shannon")+
  theme(plot.title = element_text(hjust = 0.5))

simp.sha <- data.frame(simpson = Simpson,group= group)
pic2 <- ggboxplot(simp.sha,
                  x="group",
                  y="simpson",
                  color="group",
                  palette = "jama", legend = "right",
                  add = "jitter",xlab="Group",ylab="alpha diversity",title="Simpson")+
  theme(plot.title = element_text(hjust = 0.5))

isimp.sha <- data.frame(isimpson = Inverse.Simpson,group= group)
pic3 <- ggboxplot(isimp.sha,
                  x="group",
                  y="isimpson",
                  color="group",
                  palette = "jama",legend = "right",
                  add = "jitter",xlab="Group",ylab="alpha diversity",title="Inverse.Simpson")+
  theme(plot.title = element_text(hjust = 0.5))+
  theme(axis.text = element_text(size = 13), axis.title = element_text(size = 15), legend.text = element_text(size = 11))
g123 <- ggarrange(pic1,pic2,pic3,nrow=1,common.legend = TRUE)
```

```
g123
```

- beta diversity

```
df_pca <- prcomp(eBay.comps_sub) 
df_pcs <-data.frame(df_pca$x, group =  group)  

percentage<-round(df_pca$sdev / sum(df_pca$sdev) * 100,2)
percentage<-paste(colnames(df_pcs),"(", paste(as.character(percentage), "%", ")", sep=""))
df_pcs$group <- as.factor(group)
library(RColorBrewer)
col <- brewer.pal(8,"Accent")[c(2,3)]
ggplot(df_pcs,aes(x=PC1,y=PC2,color=group,shape=group)) + geom_point(size=2)+
  xlab(percentage[1]) + ylab(percentage[2]) + 
  scale_fill_manual(values=col)+
  scale_colour_manual(values=col)+
  scale_shape_manual(values = c(2, 9))+
  theme_classic()
```

### 3.5 Differential abundance analysis: An illustration

To illustrate the usefulness of the empirical Bayes method, we apply it to the COMBO dataset for differential abundance testing. We categorize the covariate BMI as normal weight, overweight, and obese, and focus on the normal weight and obese individuals. There are 70 individuals and 62 OTUs.

```
meta_sam <- metadata %>% .[.$bmicat1norm2ow3ob!=2, ]
comps_clr_sub <- comps_clr[as.character(meta_sam$pid), ]

comps_test <- apply(comps_clr_sub, 2, function(x) { t.test(x[meta_sam$bmicat1norm2ow3ob==1],
                                                           x[meta_sam$bmicat1norm2ow3ob==3])$p.value })
dif.otus <- names(which(p.adjust(comps_test, 'fdr')<0.05))
tax.dif.otus <- tax_table(phyloseq.obj)[dif.otus, ]
# colnames(tax.dif.otus) <- "taxanomy"
datatable(tax.dif.otus)
```

We also compared eBay to other differential abundance testing methods, such as MetagenomeSeq, DESeq2, ANCOM, rarefying, total sum scaling (tss).

**Load Packages**

```
library(metagenomeSeq); 
library(DESeq2); 
source('/Users/tiantian/Downloads/ancom_functions.r')
library(UpSetR);
```

**metagenomeSeq**

```
otu_tab_sub <- otu_tab[rownames(meta_sam), ]
phenotypeData <- AnnotatedDataFrame(meta_sam)
obj_meta <- newMRexperiment(t(otu_tab_sub),phenoData=phenotypeData) %>% cumNorm(., p = 0.5)
mod_meta <- model.matrix(~meta_sam$bmicat1norm2ow3ob,data = environment(obj_meta))
res_meta <- fitFeatureModel(obj = obj_meta, mod = mod_meta)%>% MRcoefs(.,number=ncol(otu_tab_sub))
res_meta.final <- rownames(res_meta)[which(res_meta$adjPvalues<0.05)]
#####MetagenomeSeq  failed to detect any OTU###
```

**DESeq2**

```
group <- meta_sam$bmicat1norm2ow3ob
deseq_data <- DESeqDataSetFromMatrix(t(otu_tab_sub+1), DataFrame(group), ~ group)
res_deseq <- estimateSizeFactors(deseq_data) %>%estimateDispersions %>%nbinomWaldTest %>% results(.)
res_deseq.final <- rownames(res_deseq)[which(res_deseq$padj<0.05)]
```

**ANCOM**

```
res_ancom <- ANCOM(OTUdat=data.frame(otu_tab_sub,Group=group),
                 sig=0.05, multcorr=3, tau=0.02, theta=0.1, repeated=FALSE)$detected
res_ancom <- gsub('X', '', res_ancom)
```

**rarefying**

```
otu <- otu_table(as.matrix(t(otu_tab_sub)), taxa_are_rows=TRUE)
sample_data <- sample_data(meta_sam)
phyloseq.obj.sub <- phyloseq(otu, sample_data)
phyloseq.obj.rare <- rarefy_even_depth(phyloseq.obj.sub, sample.size = min(sample_sums(phyloseq.obj.sub))*0.9 ,rngseed = 2018)
res_rare <- apply(otu_table(phyloseq.obj.rare), 1, function(x) {
  t.test(x[meta_sam$bmicat1norm2ow3ob==1], x[meta_sam$bmicat1norm2ow3ob==3])$p.value
})
res_rare.final <- names(which(p.adjust(res_rare, 'BH') < 0.05))
#####rerafying  failed to detect any OTU###
```

**tss**

```
otu_tab_rep <- otu_tab_sub
otu_tab_rep[otu_tab_rep==0] <- 0.5
otu_tab_tss <- otu_tab_rep/rowSums(otu_tab_rep)
tss_clr <- apply(otu_tab_tss, 1, function(x){log2(x) - mean(log2(x))})

res_tss <- apply(tss_clr, 1, function(x) { t.test(x[meta_sam$bmicat1norm2ow3ob==1],
                                                  x[meta_sam$bmicat1norm2ow3ob==3])$p.value })
res_tss.final <- names(which(p.adjust(res_tss, 'BH') < 0.05))
```

**none**

```
res_raw <- apply(otu_tab_sub, 2, function(x) {t.test(x[meta_sam$bmicat1norm2ow3ob==1], x[meta_sam$bmicat1norm2ow3ob==3])$p.value })
res_raw.final <- names(which(p.adjust(res_raw, 'BH') < 0.05))
```

The results are shown as follows:

```
dataForUpSetPlot <- list("none-t"=res_raw.final, 
                         "tss-t"=res_tss.final, 
                         "rarefying-t"=res_rare.final, 
                        "metagenomeSeq"=res_meta.final,
                        "eBay-t"=dif.otus,
                        "eBay-tree-t"=dif.otus,
                        "DESeq2"=res_deseq.final, 
                        "ANCOM"=res_ancom)

setsBarColors <-c("dodgerblue", "goldenrod1", "darkorange1", "seagreen3", "orchid3",  'brown2', 'pink',"dodgerblue1")

res <- upset(fromList(dataForUpSetPlot),
      nsets=length(dataForUpSetPlot),
      keep.order = TRUE,
      point.size = 1,
      line.size = 0.5,
      number.angles = 0,
      shade.alpha=1,
      matrix.dot.alpha=1,
      text.scale = c(1.5, 1.2, 1.2, 1, 1.5, 1), 
      matrix.color="black",
      main.bar.color = 'black',
      mainbar.y.label = 'Intersection Size',
      sets.bar.color=setsBarColors)
```

```
res
```

## 4. Regression with relative abundances as predictors

In some machine learning applications, microbiome relative abundance data are used as predictors or inputs that have some influence on one or more outputs [8]. This section is concerned with regression problems where the predictors are compositional data and the response is univariate.

### 4.1 Constrained lasso and log-ratio lasso

To remove the unit-sum constraint, Aitchison, J. and Bacon-Shone, J. proposed a linear log-contrast model [9]. Lin, W. et al. proposed constrained lasso for fitting this model in high dimensions [10]. To select a sparse collection of log-contrasts, Bates, S. and Tibshirani, R. introduced the all-pairs log-ratio model [11]. They proposed a two-step procedure for finding a highly sparse solution, which is implemented in the R `logratiolasso` package. To illustrate these two methods, consider the regression of BMI on the estimated microbiome compositions (see Estimating microbiome compositions).

**Load Packages**

```
library(logratiolasso) # An R implementation of the constrained lasso and two-stage logratio lasso.
source("/Users/tiantian/Downloads/plot_onTree.R")
```

```
y <- metadata$bmi
x <- eBay.comps %>% log2 # log of estimated microbiome compositions

centered_y <- y - mean(y)
centered_x <- scale(x, center=T, scale=F)

# constrained lasso 
constrain_lasso <- glmnet.constr(centered_x, centered_y, family = 'gaussian')
set.seed(10)
cv_constrain_lasso <- cv.glmnet.constr(constrain_lasso, centered_x, centered_y) 
# str(cv_constrain_lasso),
# two-stage log-ratio lasso
set.seed(10)
cv_ts_lasso <- cv_two_stage(centered_x, centered_y, k_max = 7) # str(cv_ts_lasso)
```

```
## [1] "Starting CV fold 1"
## [1] "Starting CV fold 2"
## [1] "Starting CV fold 3"
## [1] "Starting CV fold 4"
## [1] "Starting CV fold 5"
## [1] "Starting CV fold 6"
## [1] "Starting CV fold 7"
## [1] "Starting CV fold 8"
## [1] "Starting CV fold 9"
## [1] "Starting CV fold 10"
```

**Plots of the CV estimates of error**

```
gl1 <- ggplot(data=NULL, aes(x=cv_constrain_lasso$lambda, cv_constrain_lasso$cvm)) + 
  geom_point() + geom_line() + labs(title='constrained lasso', x='lambda', y='CV error')+ theme_sets +
  geom_vline(xintercept = cv_constrain_lasso$lambda[which.min(cv_constrain_lasso$cvm)], 
             color='red', linetype="dotted")
gl2 <- ggplot(data=NULL, aes(x=cv_ts_lasso$lambda, y=cv_ts_lasso$mse[cv_ts_lasso$k_min,])) +
  geom_point() + geom_line() + labs(title='two-stage log-ratio lasso', x='lambda', y='CV error') +
  theme_sets +
  geom_vline(xintercept = cv_ts_lasso$lambda[cv_ts_lasso$lambda_min], color='red', 
             linetype="dotted")

gl12 <- ggarrange(gl1, gl2, nrow=1)
```

```
gl12
```

**View estimated coefficients**

```
beta12 <- data.frame(b1=cv_constrain_lasso$beta, b2=cv_ts_lasso$beta_min)
colnames(beta12) <- c('constrained lasso',  'two-stage log-ratio lasso')
datatable(round(beta12[rowSums(beta12)!=0, ], 4))
```

### 4.2 Phylogeny-aware subcomposition selection

Since a composition carries only relative information, subcompositions, which preserve ratio relationships, are fundamental objects of investigation in compositional data analysis. In the regression setting, Wang, T. and Zhao, H. proposed a multiscale method, Tree-guided Automatic Subcomposition Selection Operator (**TASSO**), for selecting subcompositions at subtree levels [12]. The criterion for TASSO contains two penalty terms corresponding to leaf nodes and internal nodes, respectively. The TASSO solution is obtained by solving a generalized lasso problem [13].

**Load Packages**

```
library(genlasso)
```

```
# constrained lasso (genlasso)
fit1_tasso <- TASSO(y,eBay.comps,tree=NULL)

# TASSO
fit2_tasso <- TASSO(y, eBay.comps,tree)
#str(fit2_tasso)
```

**Tuning via BIC**

```
# BIC for constrained lasso and TASSO
fit1_tasso_IC <- ICgenlasso(fit1_tasso)
fit2_tasso_IC <- ICgenlasso(fit2_tasso)

gf1 <- ggplot(data=fit1_tasso_IC, aes(x=lambda, y=BIC)) + geom_line() +
  geom_point()  + theme_sets + labs(title='constrained lasso (genlasso)') +
  geom_vline(xintercept = fit1_tasso_IC$lambda[which.min(fit1_tasso_IC$BIC)], color='red', linetype='dotted')
gf2 <- ggplot(data=fit2_tasso_IC, aes(x=lambda, y=BIC)) + 
  geom_point() + geom_line() + theme_sets + labs(title='TASSO') +
  geom_vline(xintercept = fit2_tasso_IC$lambda[which.min(fit2_tasso_IC$BIC)], color='red', linetype='dotted')
gf12 <- ggarrange(gf1, gf2, nrow=1)
```

```
gf12
```

**View estimated coefficients**

```
fit1_beta <- fit1_tasso$beta[, which.min(fit1_tasso_IC$BIC)] 
fit1_beta <- round(fit1_beta - mean(fit1_beta) , 8)
fit2_beta <- fit2_tasso$beta[, which.min(fit2_tasso_IC$BIC)]
fit2_beta <- round(fit2_beta - mean(fit2_beta) , 8)


tasso_beta12 <- data.frame(b1=beta12[,1], b2=fit1_beta, b3=beta12[,2], b4=fit2_beta)
colnames(tasso_beta12) <- c('constrained lasso', 'constrained lasso (genlasso)', 'two-stage log-ratio lasso', 'TASSO')
rownames(tasso_beta12) <- taxa_names(phyloseq.obj)
tasso_beta12_nonzero <- tasso_beta12[rowSums(tasso_beta12)!=0, ]
datatable(round(tasso_beta12_nonzero, 4))
```

**Visualize the TASSO results on the tree**

```
plot_onTree(fit2_tasso, tree=tree, model = 'TASSO')
```

### 4.4 Linear regression and variable fusion

So far compositions are assumed to lie in a strictly positive simplex. The main reason is that we cannot take the logarithm of zero in log-contrasts or log-ratios. To take into account the compositional nature, high dimensionality, and phylogeny of microbiome data, Wang, T. and Zhao, H. introduced the concept of variable fusion and proposed a multiscale dimension reduction method [14]. Instead of using linear log-contrast model, they used linear model. For increased interpretability, Wang, T. and Zhao, H. proposed tree-guided variable fusion to harness a predictive microbial signature made of a set of multi-level taxa. They constructed two weighted fused lasso penalties that encode the tree topology. Again, the optimization problem has a generalized lasso formulation.

```
fit_tflasso1 <- TreeFusedlasso(y, eBay.comps, tree)
fit_tflasso1
```

```
## 
## Call:
## genlasso(y = centered_Y, X = centered_X, D = D.mat.w)
## 
## Output:
## Path model with 153 total steps.
```

```
# fit_tflasso2 <- TreeFusedlasso(y, eBay.comps, tree, type = 2)
# fit_tflasso2
```

**Tuning via BIC**

```
tgfused_lasso_IC1 <- ICgenlasso(fit_tflasso1)
# tgfused_lasso_IC2 <- ICgenlasso(fit_tflasso2)
# datatable(round(tgfused_lasso_IC2, 5)) 

gf3 <- ggplot(tgfused_lasso_IC1, aes(x=lambda, y=BIC)) + 
  geom_point() + geom_line() + theme_sets + labs(title='tree-guided fused lasso penalty 1') +
   geom_vline(xintercept = tgfused_lasso_IC1$lambda[which.min(tgfused_lasso_IC1$BIC)], color='red', linetype='dotted')
# gf4 <- ggplot(tgfused_lasso_IC2, aes(x=lambda, y=BIC)) + 
#   geom_point() + geom_line() + theme_sets + labs(title='tree-guided fused lasso penalty 2') +
#   geom_vline(xintercept = tgfused_lasso_IC2$lambda[which.min(tgfused_lasso_IC2$BIC)], color='red', linetype='dotted')
# gf34 <- ggarrange(gf3, gf4, nrow=1, ncol=2)
```

```
gf3 # both the biggest lambda get the smallest BIC
```

**Visualize the results on the tree**

```
plot_onTree(fit_tflasso1, tree=tree, model = 'TreeFusedlasso')
```

## Reference

[1] : Wu, G.D., Chen, J., Hoffmann, C., Bittinger, K., Chen, Y.-Y., Keilbaugh, S.A., et al.: Linking long-term dietary patterns with gut microbial enterotypes. Science 334(6052), 105–108 (2011)

[2] : Kim, Juhyun, et al. “MGLM: An R package for multivariate categorical data analysis.” The R Journal 10.1 (2018): 73.

[3] : Wang, T., Zhao, H.: A Dirichlet-tree multinomial regression model for associating dietary nutrients with gut microorganisms. Biometrics 73(3), 792–801 (2017)

[4] : Zhou, C., Zhao, H., and Wang, T. (2021). Transformation and differential abundance analysis of microbiome data incorporating phylogeny. Bioinformatics 37(24), 4652-4660 (2021)

[5] : McMurdie, P.J., Holmes, S.: Waste not, want not: Why rarefying microbiome data is inadmissible. PLoS Computational Biology 10(4), e1003531 (2014)

[6] : Liu, T., Zhao, H. & Wang, T. An empirical Bayes approach to normalization and differential abundance testing for microbiome data. BMC Bioinformatics 21, 225 (2020).

[7] : Gloor, G.B., Macklaim, J.M., Pawlowsky-Glahn, V., Egozcue, J.J.: Microbiome datasets are compositional: And this is not optional. Frontiers in Microbiology 8, 1–6 (2017)

[8] : Knights, D., Parfrey, L.W., Zaneveld, J., Lozupone, C., Knight, R.: Human-associated microbial signatures: Examining their predictive value. Cell Host & Microbe 10(4), 292–296 (2011)

[9] : Aitchison, J., Bacon-Shone, J.: Log contrast models for experiments with mixtures. Biometrika 71(2), 323–330 (1984)

[10] : Lin, W., Shi, P., Feng, R., Li, H.: Variable selection in regression with compositional covariates. Biometrika 104(4), 785–797 (2014)

[11] : Bates, S., Tibshirani, R.: Log-ratio lasso: Scalable, sparse estimation for log-ratio models. Biometrics 75 (2), 613–624 (2019)

[12] : Wang, T., Zhao, H.: Structured subcomposition selection in regression and its application to microbiome data analysis. The Annals of Applied Statistics 11(2), 771–791 (2017)

[13] : Tibshirani, R.J., Taylor, J.: The solution path of the generalized lasso. The Annals of Statistics 39(3), 1335–1371 (2011)

[14] : Wang, T., Zhao, H.: Constructing predictive microbial signatures at multiple taxonomic levels. Journal of the American Statistical Association 112(519), 1022–1031 (2017)

```
sessionInfo()
```

```
## R version 4.1.0 (2021-05-18)
## Platform: x86_64-apple-darwin17.0 (64-bit)
## Running under: macOS Mojave 10.14.5
## 
## Matrix products: default
## BLAS:   /Library/Frameworks/R.framework/Versions/4.1/Resources/lib/libRblas.dylib
## LAPACK: /Library/Frameworks/R.framework/Versions/4.1/Resources/lib/libRlapack.dylib
## 
## locale:
## [1] zh_CN.UTF-8/zh_CN.UTF-8/zh_CN.UTF-8/C/zh_CN.UTF-8/zh_CN.UTF-8
## 
## attached base packages:
## [1] stats4    parallel  stats     graphics  grDevices utils     datasets 
## [8] methods   base     
## 
## other attached packages:
##  [1] logratiolasso_0.1.0         UpSetR_1.4.0               
##  [3] DESeq2_1.32.0               SummarizedExperiment_1.22.0
##  [5] MatrixGenerics_1.4.3        matrixStats_0.61.0         
##  [7] GenomicRanges_1.44.0        GenomeInfoDb_1.28.4        
##  [9] IRanges_2.26.0              S4Vectors_0.30.2           
## [11] metagenomeSeq_1.34.0        glmnet_4.1-3               
## [13] limma_3.48.3                Biobase_2.52.0             
## [15] BiocGenerics_0.38.0         RColorBrewer_1.1-2         
## [17] vegan_2.5-7                 lattice_0.20-45            
## [19] permute_0.9-7               ggpubr_0.4.0               
## [21] DT_0.21                     phyloMDA_0.1.0             
## [23] adaANCOM_0.2.1              ggtree_3.0.4               
## [25] phyloseq_1.36.0             BiocManager_1.30.16        
## [27] readxl_1.3.1                dplyr_1.0.8                
## [29] ggplot2_3.3.5               miLineage_2.1              
## [31] geepack_1.3.3               data.table_1.14.2          
## [33] foreach_1.5.2               magrittr_2.0.2             
## [35] genlasso_1.5                igraph_1.2.11              
## [37] Matrix_1.4-1                caper_1.0.1                
## [39] mvtnorm_1.1-3               MASS_7.3-55                
## [41] ape_5.6-1                   plyr_1.8.6                 
## [43] MGLM_0.2.0                 
## 
## loaded via a namespace (and not attached):
##   [1] backports_1.4.1        lazyeval_0.2.2         splines_4.1.0         
##   [4] BiocParallel_1.26.2    crosstalk_1.2.0        digest_0.6.29         
##   [7] yulab.utils_0.0.4      htmltools_0.5.2        fansi_1.0.2           
##  [10] memoise_2.0.1          cluster_2.1.2          annotate_1.70.0       
##  [13] Biostrings_2.60.2      colorspace_2.0-3       blob_1.2.2            
##  [16] xfun_0.29              crayon_1.5.0           RCurl_1.98-1.6        
##  [19] jsonlite_1.8.0         genefilter_1.74.1      survival_3.3-0        
##  [22] iterators_1.0.14       glue_1.6.2             gtable_0.3.0          
##  [25] zlibbioc_1.38.0        XVector_0.32.0         DelayedArray_0.18.0   
##  [28] car_3.0-12             Rhdf5lib_1.14.2        shape_1.4.6           
##  [31] abind_1.4-5            scales_1.1.1           DBI_1.1.2             
##  [34] rstatix_0.7.0          Rcpp_1.0.8.3           xtable_1.8-4          
##  [37] gridGraphics_0.5-1     tidytree_0.3.8         bit_4.0.4             
##  [40] httr_1.4.2             htmlwidgets_1.5.4      gplots_3.1.1          
##  [43] ellipsis_0.3.2         XML_3.99-0.9           pkgconfig_2.0.3       
##  [46] farver_2.1.0           sass_0.4.0             locfit_1.5-9.4        
##  [49] utf8_1.2.2             AnnotationDbi_1.54.1   ggplotify_0.1.0       
##  [52] tidyselect_1.1.2       labeling_0.4.2         rlang_1.0.1           
##  [55] reshape2_1.4.4         cachem_1.0.6           munsell_0.5.0         
##  [58] cellranger_1.1.0       tools_4.1.0            cli_3.2.0             
##  [61] RSQLite_2.2.10         generics_0.1.2         ade4_1.7-18           
##  [64] broom_0.7.12           evaluate_0.15          biomformat_1.20.0     
##  [67] stringr_1.4.0          fastmap_1.1.0          yaml_2.3.5            
##  [70] bit64_4.0.5            knitr_1.37             caTools_1.18.2        
##  [73] purrr_0.3.4            KEGGREST_1.32.0        nlme_3.1-155          
##  [76] aplot_0.1.2            compiler_4.1.0         rstudioapi_0.13       
##  [79] png_0.1-7              ggsignif_0.6.3         treeio_1.16.2         
##  [82] geneplotter_1.70.0     tibble_3.1.6           bslib_0.3.1           
##  [85] stringi_1.7.6          highr_0.9              ggsci_2.9             
##  [88] multtest_2.48.0        vctrs_0.3.8            pillar_1.7.0          
##  [91] lifecycle_1.0.1        rhdf5filters_1.4.0     jquerylib_0.1.4       
##  [94] cowplot_1.1.1          bitops_1.0-7           patchwork_1.1.1       
##  [97] R6_2.5.1               KernSmooth_2.23-20     gridExtra_2.3         
## [100] codetools_0.2-18       exactRankTests_0.8-34  gtools_3.9.2          
## [103] assertthat_0.2.1       Wrench_1.10.0          rhdf5_2.36.0          
## [106] withr_2.5.0            GenomeInfoDbData_1.2.6 mgcv_1.8-39           
## [109] grid_4.1.0             ggfun_0.0.5            tidyr_1.2.0           
## [112] rmarkdown_2.12         carData_3.0-5
```
